# Impact of Integrated Care Management on Clinical Outcomes in Atrial Fibrillation Patients: A Report From the FANTASIIA Registry

**DOI:** 10.3389/fcvm.2022.856222

**Published:** 2022-05-02

**Authors:** María Asunción Esteve-Pastor, Martín Ruiz-Ortiz, Javier Muñiz, Inmaculada Roldán-Rabadán, Déborah Otero, Ángel Cequier, Vicente Bertomeu-Martínez, Lina Badimón, Manuel Anguita, Gregory Y. H. Lip, Francisco Marín

**Affiliations:** ^1^Department of Cardiology, Hospital Clínico Universitario Virgen de la Arrixaca, CIBERCV, Instituto Murciano de Investigación Biosanitaria (IMIB-Arrixaca), Murcia, Spain; ^2^Department of Cardiology, Hospital Universitario Reina Sofía, Córdoba, Spain; ^3^Grupo de Investigación Cardiovascular, Departamento de Ciencias de la Salud e Instituto de Investigación Biomédica de A Coruña (INIBIC), CIBERCV, Universidade da Coruña, A Coruña, Spain; ^4^Department of Cardiology, Hospital La Paz, Madrid, Spain; ^5^ODDS, SL, A Coruña, Spain; ^6^Department of Cardiology, Hospital de Bellvitge, CIBERCV, Barcelona, Spain; ^7^Department of Cardiology, Hospital Universitario de San Juan, Alicante, Spain; ^8^Cardiovascular Research Center (CSIC-ICCC), Hospital de la Santa Creu i Sant Pau, CIBERCV, IIB-Sant Pau, Barcelona, Spain; ^9^Liverpool Centre for Cardiovascular Science, University of Liverpool and Liverpool Heart and Chest Hospital, Liverpool, United Kingdom; ^10^Department of Clinical Medicine, Aalborg University, Aalborg, Denmark

**Keywords:** atrial fibrillation, patient adherence, risk factors, ABC-pathway, integrated approach

## Abstract

**Background:**

An integrated and holistic approach is increasingly advocated in patients with atrial fibrillation (AF), based on the “Atrial fibrillation Better Care (ABC) pathway: A, Avoid stroke with anticoagulation; B, better symptom management; C, cardiovascular and comorbidity risk management.” The aim of this study was to examine the prevalence of adherence to each component of the ABC pathway and to analyze its impact on long-term prognosis in the “real-world” cohort of AF patients from the FANTASIIA registry.

**Methods:**

This prospective study included consecutive AF outpatients anticoagulated with direct oral anticoagulants (DOAC) or vitamin K antagonists (VKA) from June 2013 to October 2014. From the ABC pathway, adherence to the “A criterion” was defined by a time in the therapeutic range (TTR) ≥ 70% or correct dose with DOAC; “B criterion” adherence was defined by a European Heart Rhythm Association (EHRA) Symptom Scale I-II; and “C criterion” adherence was defined as optimized risk factors and comorbidity management. Baseline features and embolic events, severe bleeding, and all-cause and cardiovascular mortality rates up to 3 years of follow-up were analyzed, and a Cox multivariate analysis was performed to investigate the role of each component of the ABC pathway in predicting major events.

**Results:**

A total of 1,955 AF patients (age: 74.4 ± 9.4 years; 43.2% female patients) were included in this study: adherence to A criterion was observed in 920 (47.1%) patients; adherence to B criterion was observed in 1,791 (91.6%) patients; and adherence to C criterion was observed in 682 (34.8%) patients. Only 394 (20.2%) of the whole population had good control of AF according to the ABC pathway. After a median follow-up of 1,078 days (IQR: 766–1,113), adherence to A criterion was independently associated with reduced cardiovascular mortality [HR: 0.67, 95%CI (0.45–0.99); *p* = 0.048] compared with non-adherence. Adherence to the B criterion was independently associated with reduced stroke [HR: 0.28, 95%CI (0.14–0.59); *p* < 0.001], all-cause mortality [HR: 0.49, 95%CI (0.35–0.69); *p* < 0.001], cardiovascular mortality [HR: 0.39, 95%CI (0.25–0.62); *p* < 0.001], and major adverse cardiovascular events (MACE) [HR: 0.41, 95%CI (0.28–0.62); *p* < 0.001] compared with non-adherence. AF patients with C criterion adherence had a significantly lower risk of myocardial infarction [HR: 0.31, 95%CI (0.15–0.66); *p* < 0.001]. Fully adherent ABC patients had a significant reduction in MACE [HR: 0.64, 95%CI (0.42–0.99); *p* = 0.042].

**Conclusion:**

In real-world anticoagulated AF patients from FANTASIIA registry, we observed a lack of adherence to integrated care management of AF following the ABC pathway. AF managed according to the ABC pathway was associated with a significant reduction in adverse outcomes during long follow-up, suggesting the benefit of a holistic and integrated approach to AF management.

## Introduction

Atrial fibrillation (AF) has emerged as a major public health issue, and more than two-thirds of patients older than 70 years will present with this disease, increasing hospital admissions and direct and indirect healthcare costs (1). The presence of AF increases the risk of stroke, cardiovascular events, and mortality (2).

Both hospitalization and complications related with AF could be preventable with appropriate guideline-adherent care to reduce adverse outcomes in those patients (3). The concept of integrated care was developed by Wagner et al. (4) regarding the effective interventions for patients with chronic diseases highlighting five areas, namely, the use of evidence-based therapies with planned care of interventions, reorganization of health systems, improved patient self-management support, increased access to expertise with multidisciplinary teams, and greater availability of clinical information (3, 4). Such an integrated care approach can be extended to various clinical settings (5, 6). In the case of AF, such an integrated and holistic approach is essential. AF management not only involves oral anticoagulation for stroke prevention but also needs an improvement of AF symptomatology with rate or rhythm control, management of risk factors with targeted treatments, or control of concomitant comorbidities to mitigate treatment burden (7, 8). Indeed, a multidisciplinary holistic approach to manage AF, including behavior changes and close follow-up, is associated with significantly lower cardiovascular outcomes and reduced medical costs (9, 10).

Such an integrated and holistic approach for the management of AF disease can be based on the “Atrial fibrillation Better Care” (ABC) pathway (11). The “A criterion” refers to Avoid stroke, i.e., identifying those patients who are truly at low risk, who do not need oral anticoagulation, and who do not use effective oral anticoagulation. When vitamin K antagonists (VKA) are used, a high time in therapeutic range (TTR) ≥ 70% is necessary for best effectiveness and safety of oral anticoagulation with VKA (12) or a label-adherent correct dose of direct oral anticoagulants (DOACs). The “B criterion” refers to patient-centered symptom-directed decisions on rate or rhythm control; and the “C criterion” refers to control of cardiovascular risk factors and comorbidities, including lifestyle changes (11, 13).

The aim of this study was to examine the prevalence of adherence to each component of the ABC pathway and to analyze its impact on long-term prognosis in the “real-world” cohort of AF patients from the FANTASIIA registry.

## Materials and Methods

The study design of the FANTASIIA registry has been described earlier (14). FANTASIIA (Spanish Acronym for “Fibrilación Auricular: influencia del Nivel y Tipo de Anticoagulación Sobre la Incidencia de Ictus y Accidentes hemorrágicos”) registry is an observational, prospective, national, and multicenter study of clinical and demographic characteristics of Spanish AF patients. In brief, the main objective of this study is to assess the incidence of thromboembolic and bleeding events in an unselected population of patients with AF, specifically, the type of oral anticoagulant (VKA or DOACs) and quality of anticoagulation with VKAs. We excluded AF patients with valvular heart disease. When we refer to AF patients throughout the article, we analyzed non-valvular atrial fibrillation patients, but that term is deprecated according to the latest 2020 ESC clinical guidelines (12) because it can be confusing. For that reason, we only write “AF patients.”

### Study Population

Between June 2013 and March 2014, all outpatients with a confirmed diagnosis of paroxysmal, persistent, or permanent AF were prospectively enrolled. Patients included in the registry had been receiving OAC (VKA or DOACs) for at least 6 months before enrollment. The study was conducted in 50 outpatient clinics by 80 investigators. To “simulate” true DOAC use during that period in Spain, in each participating center, 1 patient receiving DOACs was included for every 4 patients receiving VKAs (predefined ratio in the protocol, 1:4 for DOACs and VKAs). Each investigator had to include the first 20 consecutive AF patients who met the inclusion and exclusion criteria (first 4 with DOACs and first 16 with VKAs).

Patients with valvular heart disease (rheumatic valve disease, moderate-severe valve disease, prosthesis or valve repair surgery), younger than 18 years old, or with recent hospital admission were excluded. All subjects provided signed informed consent. The study was conducted according to the ethical principles of the Declaration of Helsinki and Good Clinical Practice Guidelines and was approved by the Clinical Research Ethics Committee at Hospital Universitario de San Juan (Spain) with the approval number 12/220 and by the Spanish Agency of Medicine and Health Products as a prospective follow-up post-authorization study with the approval number SEC-ACO-2012-01.

### Data Collection

Clinical and demographic data for all AF patients were collected in a detailed medical history. Blood sample was collected at baseline and in each visit, and we collected some variables such as glucose, hemoglobin, creatinine, lipid profile, or HbA1c (hemoglobin A1c). We also collected all drug therapy of the patient at baseline. Coagulation status was determined by the International normalized ratio (INR) values of the 6 months prior to the study entry. The estimated time spent in the therapeutic range (TTR) was assessed by the Rosendaal method. Stroke risk was calculated using the CHADS_2_ (15) (Congestive heart failure, Hypertension, Age ≥ 75 years, Diabetes mellitus, Stroke or transient ischemic attack [TIA]) and CHA_2_DS_2_-VASc scores (16) [Congestive heart failure, Hypertension, Age ≥ 75 years, Diabetes mellitus, Stroke or transient ischemic attack, Vascular disease, Age 65–74 years and Sex category (female)]. Bleeding risk was calculated using the HAS-BLED score (17) [Hypertension (uncontrolled systolic blood pressure > 160 mmHg), Abnormal renal and/or liver function, previous Stroke, Bleeding history or predisposition (anemia), Labile INR (only applies to a VKA user; not applicable for a non-VKA user), Elderly (age ≥ 65 years), and concomitant Drugs (antiplatelet or non-steroidal anti-inflammatory drugs) and/or alcohol excess].

### Definitions

Following the latest clinical guidelines and ABC pathway definitions (11), the A, B, and C components for this analysis were defined as follows (Supplementary Figure 1):

• Adherence to the “**A component**” was defined as “avoid stroke/high quality of oral anticoagulation.” For VKA users, this was fulfilled if TTR ≥ 70%. As all our patients were under oral anticoagulation therapy at enrollment, for DOAC users we use the definition of Pastori et al. (18); this was the use of appropriate label-adherent dose, as defined following the EHRA practical guide (19). For patients receiving dabigatran, criteria for considering reduced dose were: age ≥ 80 years; concomitant use of verapamil; ≥ 2 of the following: age 75–79 years, creatinine clearance (CrCl) 30–50 ml/min, HAS-BLED ≥ 3, amiodarone use, platelet aggregation inhibitors use, or body weight ≤ 60 kg. For patients receiving rivaroxaban, criteria for considering reduced dose were: CrCl 15–49 ml/min; ≥ 2 of the following: age ≥ 75 years, HAS-BLED ≥ 3, amiodarone use, platelet aggregation inhibitors use, or body weight ≤ 60 kg. For patients receiving apixaban, criteria for considering reduced dose were: ≥ 2 of the following: age ≥ 80 years, creatinine ≥ 1.5 mg/dl, or body weight ≤ 60 kg; CrCl 15–29 ml/min; ≥ 2 of the following: age ≥ 75 years, HAS-BLED ≥ 3, amiodarone use, platelet aggregation inhibitors use, or diltiazem use. We had no patients taking edoxaban therapy in our registry.
• Adherence to the “**B component**” refers to better AF symptom management. We used a validated method, i.e., the modified European Heart Rhythm Association (EHRA) score was used (20). EHRA Class I refers to asymptomatic AF disease; EHRA Class II refers to mild symptoms and normal daily activity not affected; EHRA Class III refers to severe symptoms that modified normal daily activity; and EHRA IV refers to disabling symptoms that result in the discontinuation of normal daily activity. We defined “well-controlled AF symptoms” if patients had EHRA I-II classification.
• Adherence to the “**C component**” refers to “Cardiovascular risk factor and comorbidity optimization.” We used strict criteria for the management of concomitant diseases according to the international guidelines. We considered the following comorbidities and diseases: hypertension, diabetes mellitus, heart failure (HF), coronary artery disease (CAD), peripheral artery disease (PAD), and stroke/TIA. For hypertension, we consider optimal management if blood pressure is < 140/90 mmHg. Optimal management of diabetes was defined as HbA1c ≤ 7.5%. If HBA_1_C values were not available, we consider well-controlled diabetic patients if fasting glucose levels were ≤ 126 mg/dl. For patients with HF, we consider as optimal management of the disease if treated with beta-blockers or diuretics or ACEI/ARBs (angiotensin-converting enzyme inhibitors/angiotensin receptor blockers). Optimal management of HF patients were consiedered if the patients were treated with two of those drugs. For patients with CAD, PAD, or stroke/TIA, we considered as well-managed of those entities if LDL ≤ 70 mg/dl [as per previous ESC guidelines (21)] and if the patients were treated with statins and with ACEI/ARBs.

Following each component individually, we defined the “ABC pathway adherent group” if all components were fulfilled. If not, the AF disease was considered as non-optimally managed according to the ABC criteria (non-ABC group).

### Clinical Outcomes

After 3 years of follow-up, we analyzed adverse clinical outcomes according to each ABC criterion fulfilled and the “ABC pathway adherent group.” Thromboembolic events were defined as stroke or transient ischemic attack or peripheral embolism. Bleeding events were assessed according to the 2005 International Society of Thrombosis and Hemostasis criteria (20): fatal bleeding or symptomatic bleeding in a critical anatomical site (intracranial, intraspinal, intraocular, retroperitoneal, intraarticular, pericardial or intramuscular with compartment syndrome) and/or bleeding causing a fall in Hb ≥ 20 g/L, or transfusion of ≥ 2 units of packed red blood cells. We also collected all-cause mortality, and cardiovascular mortality was defined if it was secondary to a cardiovascular event (acute coronary syndrome, heart failure, lethal arrhythmia or sudden death, artery aneurysm rupture or stroke). MACE (major adverse cardiovascular events) was defined as the composite of ischemic stroke, myocardial infarction, and cardiovascular mortality. Cardiovascular events included fatal and non-fatal stroke, TIA, myocardial infarction, coronary revascularization, and cardiovascular mortality. Clinically significant events included the composite of stroke, major bleeding, all-cause mortality, acute coronary syndrome, and acute heart failure. An external event assignment committee evaluated all adverse events.

### Statistical Analysis

Normal distribution of continuous variables was tested with the Kolmogorov-Smirnov method. Continuous variables are presented using the mean ± standard deviation or median [interquartile range]. Categorical variables are expressed as percentages. For between-group comparisons, we used the Student’s *t*-test for continuous variables and chi-square test for qualitative variables. Cox regression analyses were used to determine the associations between adverse events and each component fulfilled individually (“A” criterion, “B” criterion, and “C” criterion) and for “ABC pathway adherent group.” The independent effect of clinical variables on adverse clinical outcomes was calculated using a Cox proportional hazards regression, including in the multivariate model only those values with *p* < 0.15 on univariate analysis. The models included the ABC components, age, sex, hypertension, diabetes, dyslipidemia, coronary artery disease, heart failure, peripheral artery disease, stroke/TIA, chronic kidney disease, and Charlson Comorbidity Index. Differences in event-free survival were examined with log-rank test, and Kaplan–Meier curves were drafted accordingly. Statistical significance was defined as *p* < 0.05. Statistical analyses were performed with Stata version 12 (Stata Corporation, College Station, TX, United States).

## Results

We enrolled 2,178 patients in FANTASIIA registry; 1,648 (75.6%) patients were treated with VKA and 530 (24.3%) patients were treated with DOACs, of which 267 (50.4% of DOAC group) patients were treated with dabigatran, 190 (35.8%) patients were treated with rivaroxaban, and 73 (13.8%) patients were treated with apixaban. A total of 1,956 patients (89% of the sample) completed full follow-up. Finally, we had available data from 1,955 patients (74.4 ± 9.4 years; 43.2% female patients) for all components of the ABC pathway for this analysis.

### Atrial Fibrillation Better Care Components and Atrial Fibrillation Better Care Pathway Adherence

Of the whole cohort, 920 (47.1%) patients achieved good control of oral anticoagulation (A criterion fulfilled); 1,791 (91.6%) patients had good control of arrhythmia symptomatology (B criterion fulfilled); and 682 (34.8%) patients had good control of cardiovascular risk factors and comorbidities (C criterion fulfilled). Only 394 (20.2%) patients had good compliance and were in the “ABC pathway adherent” group.

In comparison with the ABC adherent group, patients with AF non-ABC adherent had high prevalence of cardiovascular risk factors such hypertension (83.9% vs. 66.8%; *p* < 0.001), diabetes mellitus (32.6% vs. 16.5%; *p* < 0.001), or chronic kidney disease (20.2% vs. 15.7%; *p* = 0.046). Patients with AF non-adherent to ABC pathway also had a high prevalence of heart disease (50.8% vs. 36.8%; *p* < 0.001), coronary artery disease (21.3% vs. 6.1%; *p* < 0.001), and high CHA_2_DS_2_-VASc (3.9 ± 1.6 vs. 2.9 ± 1.4; *p* < 0.001) and HAS-BLED scores (2.1 ± 1.1 vs. 1.6 ± 0.9; *p* < 0.001). No differences between sex, age and type of AF were observed (Table 1).

**TABLE 1:** Baseline characteristics of patients according to the Atrial fibrillation Better Care (ABC) pathway adherence.

Variable	ABC-pathway adherence group *n* = 394	Non-ABC group *n* = 1,561	*P*-value
Age	73.1 ± 9.8	73.9 ± 9.4	0.053
Sex (female)	168 (42.6)	692 (44.3)	0.561
**Comorbidities**			
Hypertension	263 (66.8)	1310 (83.9)	<0.001
Diabetes mellitus	65 (16.5)	509 (32.6)	<0.001
Dyslipidemia	189 (47.9)	830 (53.2)	0.062
Current smoker	22 (5.6)	76 (4.9)	0.598
COPD	57 (14.5)	286 (18.3)	0.072
PAD	3 (0.8)	116 (7.4)	<0.001
CKD	62 (15.7)	315 (20.2)	0.046
Excessive alcohol use	11 (2.8)	61 (3.9)	0.293
Charlson’s Index	0.7 ± 0.9	1.3 ± 1.2	<0.001
BMI (kg/m^2^)	28.3 ± 4.9	29.1 ± 4.8	0.009
Previous heart disease	145 (36.8)	793 (50.8)	<0.001
Heart failure	91 (23.1)	475 (30.4)	0.004
Coronary artery disease	24 (6.1)	332 (21.3)	<0.001
LVEF < 45%	41 (10.4)	186 (11.9)	0.385
LVEF	60.3 ± 10.6	58.1 ± 11.5	<0.001
Hb(g/dl)	13.8 ± 1.6	13.6 ± 1.7	0.016
eGFR	67.7 ± 22.4	65.8 ± 23.1	0.133
Previous stroke/TIA	16 (4.1)	315 (20.2)	<0.001
Previous major bleeding	9 (2.3)	72 (4.6)	0.046
**Atrial fibrillation**			
Paroxysmal AF	116 (29.4)	454 (29.1)	0.959
Persistent AF	66 (16.7)	262 (16.8)	
Permanent AF	212 (53.8)	845 (54.1)	
Electrical cardioversion	82 (20.8)	272 (17.4)	0.119
AF ablation	20 (5.1)	64 (4.1)	0.393
Rhythm control	155 (39.3)	592 (37.9)	0.605
Rate control	239 (60.7)	969 (62.1)	
**Concomitant treatment**			
Digoxin	73 (18.5)	283 (18.1)	0.831
Beta-blockers	239 (60.6)	940 (60.2)	0.873
Antiarrhythmic agents	102 (25.9)	379 (24.3)	0.525
Diuretic	201 (51.1)	921 (59.0)	0.004
ACE/ARB inhibitors	260 (65.9)	1138 (72.9)	0.461
Antiplatelet agents	20 (5.08)	189 (11.9)	<0.001
**Anticoagulant therapy**			<0.001
VKA (*n* = 1469)	257 (65.2)	1224 (78.5)	
NOACs (*n* = 467)	137 (34.8)	334 (21.4)	
**CHADS_2_**	1.6 ± 1.1	2.4 ± 1.2	<0.001
**CHA_2_DS_2_-VASc**	2.9 ± 1.4	3.9 ± 1.6	<0.001
**HAS-BLED**	1.6 ± 0.9	2.1 ± 1.1	<0.001

*COPD, Chronic obstructive pulmonary disease; PAD, peripheral artery disease; CAD, coronary artery disease; CKD, chronic kidney disease; TIA, transitory ischemic attack; LVEF, left ventricular ejection fraction; BMI (kg/m^2^), body mass index; Hb, hemoglobin; ACE, angiotensin-converting enzyme; ARB, angiotensin receptor blockers; VKA, vitamin K antagonists; NOACs, non-vitamin K oral anticoagulants; CHADS_2_ = Congestive heart failure or left ventricular dysfunction (1); Hypertension (1), Age ≥ 75 (2), Diabetes mellitus (1), prior Stroke/TIA or systemic embolism (2). CHA_2_DS_2_-VASc = Congestive heart failure or left ventricular dysfunction (1); Hypertension (1), Age ≥ 75 (2) or 65–74 (1), Diabetes mellitus (1), prior Stroke/TIA or systemic embolism (2), Vascular disease (peripheral artery disease, myocardial infarction, aortic plaque) (1), Sex category (i.e., female sex) (1); HAS-BLED = Hypertension (1), Abnormal renal and/or liver function (1), prior Stroke (1), Bleeding history or predisposition (1), Labile INR (1), Elderly (1), Drugs or excess alcohol (1).*

### Clinical Outcomes

After a median follow-up of 1,078 days (IQR: 766–1,113), we analyzed all the adverse events in the “ABC pathway adherent group” and to the adherence of each criterion. There was a total of 255 deaths (107 of them were related with cardiovascular causes), 45 strokes, and 136 major bleedings.

### “A” Criterion

**FIGURE 1:**
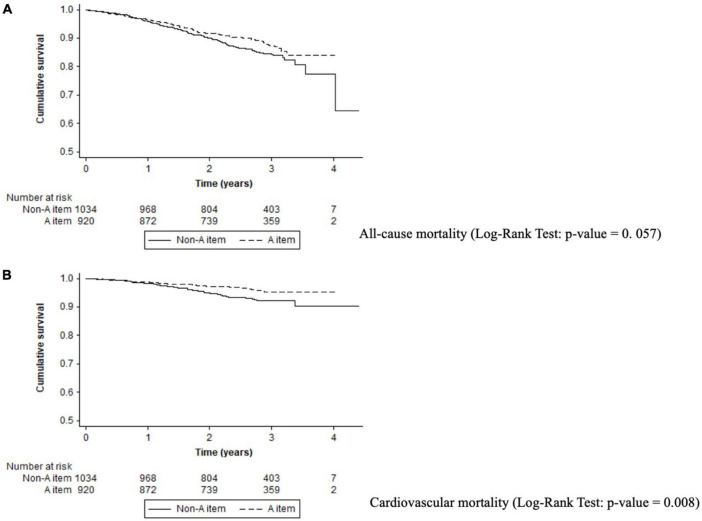
Kaplan–Meier curves for adverse events in A criterion. **(A)** All-cause mortality (log-rank test: *p*-value = 0.057). **(B)** Cardiovascular mortality (log-rank test: *p*-value = 0.008).

We observed patients with non-adherence to the “A” criterion had a higher rate of all-cause mortality (5.69%/year vs. 4.44%/year; *p* = 0.043), cardiovascular mortality (2.65%/year vs. 1.57%/year; *p* = 0.008), and cardiovascular events (3.63%/year vs. 2.73%/year) compared with adherent patients (see Table 2). Patients with AF non-adherence to the component A had a high risk of all-cause mortality (log-rank *p*-value = 0.057) and cardiovascular mortality (log-rank *p*-value = 0.008) (Figure 1).

**TABLE 2:** Distribution of adverse events and annual rates according to the compliance with each criterion and with ABC pathway.

	Compliance n; Annual rate (%/year)	Non-compliance n; Annual rate (%/year)	*P*-value
**Ischemic stroke**			
“A” criterion	20 (0.85%)	25 (0.96%)	0.720
“B” criterion	34 (0.73%)	11 (3.24%)	**<0.001**
“C” criterion	15 (0.88%)	28 (1%)	0.690
“ABC” pathway	7 (0.69%)	38 (0.96%)	0.410
**All-cause mortality**			
“A” criterion	105 (4.44%)	150 (5.69%)	**0.043**
“B” criterion	207 (4.44%)	48 (13.79%)	**<0.001**
“C” criterion	94 (5.48%)	152 (5.38%)	0.887
“ABC” pathway	43 (4.20%)	212 (5.33%)	0.156
**Cardiovascular mortality**			
“A” criterion	37 (1.57%)	70 (2.65%)	**0.008**
“B” criterion	78 (1.67%)	29 (8.33%)	**<0.001**
“C” criterion	37 (2.16%)	67 (2.37%)	0.645
“ABC” pathway	15 (1.47%)	92 (2.31%)	0.099
**Acute myocardial infarction**			
“A” criterion	25 (1.07%)	28 (1.06%)	0.980
“B” criterion	46 (1%)	7 (2.05%)	0.069
“C” criterion	8 (0.47%)	42 (1.51%)	**<0.001**
“ABC” pathway	6 (0.59%)	47 (1.19%)	0.097
**MACE**			
“A” criterion	69 (2.96%)	99 (3.81%)	0.103
“B” criterion	130 (2.83%)	38 (11.25%)	**<0.001**
“C” criterion	50 (2.94%)	112 (4.04%)	**0.045**
“ABC” pathway	24 (2.37%)	144 (3.68%)	**0.044**
**Cardiovascular events**			
“A” criterion	74 (2.73%)	111 (3.63%)	**0.043**
“B” criterion	143 (2.71%)	42 (8.63%)	**<0.001**
“C” criterion	55 (2.73%)	122 (3.74%)	**0.041**
“ABC” pathway	27 (2.32%)	158 (3.43%)	**0.048**
**Major bleeding**			
“A” criterion	68 (2.94%)	78 (3.05%)	0.898
“B” criterion	129 (2.84%)	17 (5.03%)	**0.025**
“C” criterion	44 (2.61%)	87 (3.17%)	0.263
“ABC” pathway	30 (2.99%)	116 (3.0%)	0.995
**Clinically significant events**			
“A” criterion	182 (8.10%)	231 (9.21%)	0.167
“B” criterion	347 (7.77%)	66 (20.33%)	**<0.001**
“C” criterion	137 (8.21%)	254 (9.48%)	0.176
“ABC” pathway	70 (7.04%)	343 (9.05%)	0.055

*MACE, major adverse clinical outcomes: composite of cardiovascular death, ischemic stroke, and myocardial infarction. Clinically significant events: composite of ischemic stroke, major bleeding, all-cause mortality, acute coronary syndrome, and acute heart failure.*

*“A” criterion compliance: 920 (47.95%) patients.*

*“B” criterion compliance: 1791 (91.6%) patients.*

*“C” criterion compliance: 682 (34.89%) patients.*

*“ABC pathway” compliance: 394 (20.2%) patients.*

*Bold values are regarding to statistical significance (it means p < 0.05).*

On multivariable regression analysis, “A” criterion adherence was independently related with reduced cardiovascular mortality [HR: 0.67, 95%CI (0.45–0.99); *p* = 0.048] in comparison with non-adherence (see Table 3 and Supplementary Table 1).

**TABLE 3:** Unadjusted and adjusted hazard ratios for adverse outcomes according to the criteria fulfilled from atrial fibrillation better care (ABC pathway adherent criteria) compared with non-optimally managed ABC group.

	Number of patients	Number of events	HR for event	Adjusted HR	*p*-value
**Stroke**					
“A” criteria fulfilled	920	20	0.89 (0.50–1.60); *p* = 0.700	0.94 (0.52–1.71)	0.847
“B” criteria fulfilled	1791	34	**0.23 (0.12**–**0.45); *p* < 0.001**	**0.28 (0.14**–**0.59)**	**<0.001**
“C” criteria fulfilled	682	15	0.88 (0.47–1.65); p = 0.690	1.36 (0.66–2.80)	0.410
“ABC” criteria fulfilled	394	7	0.71 (0.32–1.60); p = 0.410	1.01 (0.43–2.37)	0.924
**All-cause mortality**					
“A” criteria fulfilled	920	105	0.78 (0.61–1.0); p = 0.052	0.84 (0.65–1.08)	0.173
“B” criteria fulfilled	1791	207	**0.32 (0.24**–**0.44); *p* < 0.001**	**0.49 (0.35**–**0.69)**	**<0.001**
“C” criteria fulfilled	682	94	1.02 (0.53–1.75); *p* = 0.903	1.27 (0.98–1.64)	0.075
“ABC” criteria fulfilled	394	43	0.79 (0.57–1.10); *p* = 0.156	1.12 (0.80–1.58)	0.514
**Cardiovascular mortality**					
“A” criteria fulfilled	920	37	**0.59 (0.40**–**0.88); *p* = 0.009**	**0.67 (0.45**–**0.99)**	**0.048**
“B” criteria fulfilled	1791	78	**0.20 (0.13**–**0.31); *p* < 0.001**	**0.39 (0.25**–**0.62)**	**< 0.001**
“C” criteria fulfilled	682	37	0.91 (0.61–1.32); *p* = 0.887	1.37 (0.88–2.14)	0.167
“ABC” criteria fulfilled	394	15	0.64 (0.37–1.10); *p* = 0.099	0.98 (0.56–1.73)	0.950
**Acute myocardial infarction**					
“A” criteria fulfilled	920	25	0.99 (0.58–1.70); *p* = 0.980	1.12 (0.65–1.94)	0.672
“B” criteria fulfilled	1791	46	0.49 (0.22–1.08); *p* = 0.069	0.82 (0.35–1.91)	0.638
“C” criteria fulfilled	682	8	**0.31 (0.15**–**0.66); *p* < 0.001**	0.53 (0.24–1.18)	0.121
“ABC” criteria fulfilled	394	6	0.49 (0.21–1.16); *p* = 0.097	0.83 (0.35–1.99)	0.680
**MACE**					
“A” criteria fulfilled	920	69	0.78 (0.57–1.05); *p* = 0.104	0.87 (0.64–1.19)	0.393
“B” criteria fulfilled	1791	130	**0.25 (0.18**–**0.36); *p* < 0.001**	**0.41 (0.28**–**0.62)**	**0.001**
“C” criteria fulfilled	682	50	0.73 (0.52–1.01); *p* = 0.059	1.08 (0.75–1.56)	0.676
“ABC” criteria fulfilled	394	24	**0.64 (0.42**–**0.99); *p* = 0.042**	0.98 (0.63–1.53)	0.923

*Bold values are regarding to statistical significance (it means p < 0.05).*

### “B” Criterion

**FIGURE 2:**
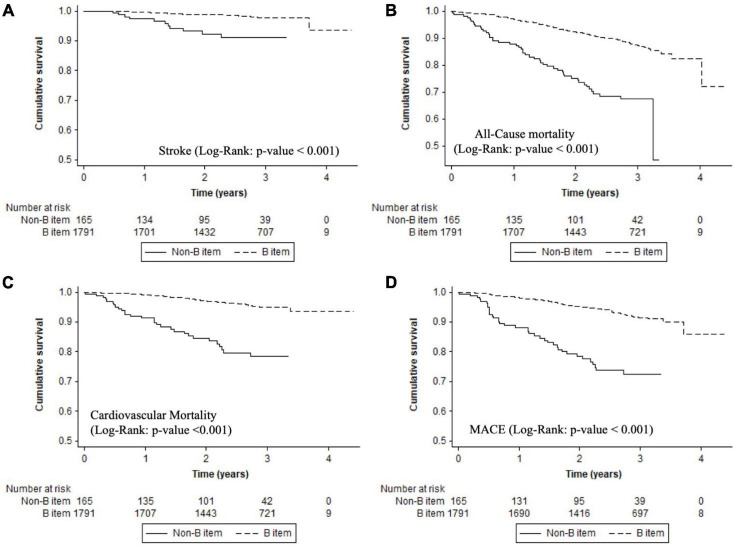
Kaplan–Meier curves for adverse events in B component. **(A)** Stroke (log-rank: *p*-value < 0.001). **(B)** All-cause mortality (log-rank: *p*-value < 0.001). **(C)** Cardiovascular mortality (log-rank: *p*-value < 0.001). **(D)** MACE (log-rank: *p*-value < 0.001).

Poor control of AF symptoms (non-adherence to B component) had higher rate of adverse events: all-cause mortality (13.79%/year vs. 4.44%/year; *p* < 0.001), cardiovascular mortality (8.33%/year vs. 1.67%/year; *p* < 0.001), MACE (11.25%/year vs. 2.83%/year; *p* < 0.001), stroke (3.24%/year vs. 0.73%/year; *p* < 0.001), or major bleeding (5.03%/year vs. 2.84%/year; *p* = 0.025) compared with patients with well-controlled AF disease, see Table 2. Patients with poor control according to the “B” criterion had high risk of stroke (log-rank *p*-value < 0.001), all-cause mortality (log-rank *p*-value < 0.001), cardiovascular mortality (log-rank *p*-value < 0.001), MACE (log-rank *p*-value < 0.001), or major bleeding (log-rank *p*-value = 0.020) (Figure 2).

On multivariable regression analysis, “B” criterion adherence was independently related with lower stroke [HR: 0.28, 95%CI

(0.14–0.59); *p* < 0.001], all-cause mortality [HR: 0.49, 95%CI (0.35–0.69); *p* < 0.001], cardiovascular mortality [HR: 0.39, 95%CI (0.25–0.62); *p* < 0.001], and MACE [HR: 0.41, 95%CI (0.28–0.62); *p* < 0.001] in comparison with non-adherence (see Table 3 and Supplementary Tables 2–5).

### “C” Criterion

Non-adherence to the C-criterion had a high rate of acute myocardial infarction (1.51%/year vs. 0.47%/year; *p* < 0.001), cardiovascular events (3.74%/year vs. 2.73%/year; *p* = 0.041), or MACE (4.04%/year vs. 2.94%/year; *p* = 0.045) compared with adherent patients (Table 2).

**FIGURE 3:**
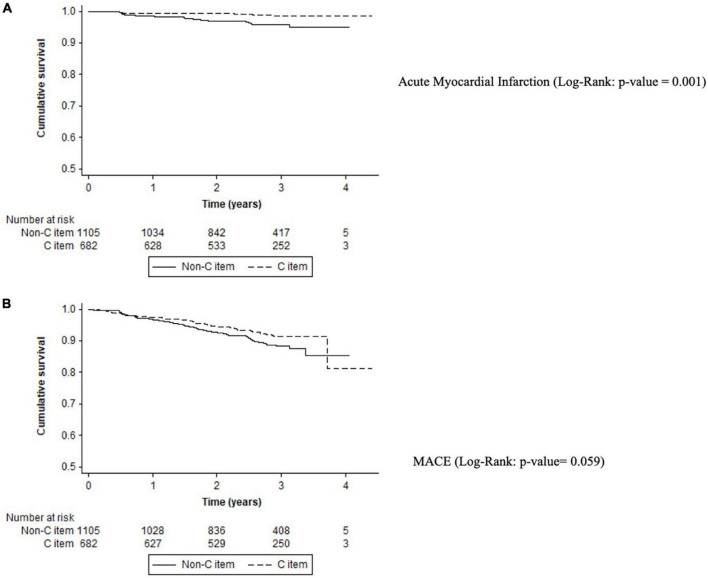
Kaplan–Meier curves for adverse events in C criterion. **(A)** Acute myocardial infarction (log-rank: *p*-value = 0.001). **(B)** MACE (log-rank: *p*-value = 0.059).

Kaplan–Meier curves show that non-adherence to C-criterion had a high risk of myocardial infarction (log-rank *p*-value < 0.001) and a trend for MACE (log-rank *p*-value = 0.059). Patients with good control of comorbidities had a significantly lower risk of myocardial infarction [HR: 0.31, 95%CI (0.15–0.66); *p* < 0.001)] (Figure 3). On the multivariable regression analysis, there was no independent association of good adherence to C criterion and adverse events (see Supplementary Table 6).

### Full “Atrial Fibrillation Better Care Pathway Adherence”

**FIGURE 4:**
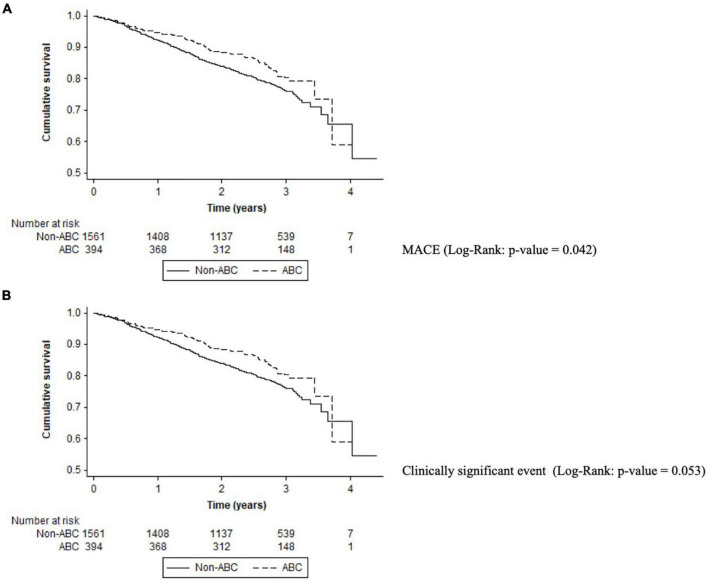
Kaplan–Meier curves for adverse events in the ABC pathway adherent group. **(A)** MACE (log-rank: *p*-value = 0.042). **(B)** Clinically significant event (log-rank: *p*-value = 0.053).

Only 394 (20.2%) patients had full adherence to all the ABC components. Patients of the non-ABC group had a higher rate of MACE (3.68%/year vs. 2.37%/year; *p* = 0.044) and cardiovascular events (3.43%/year vs. 2.32%/year; *p* = 0.048) compared with ABC pathway adherent patients. Adherent ABC patients had a significant reduction in MACE [HR: 0.64, 95%CI (0.42–0.99); *p* = 0.042). All adverse events according to the ABC pathway are shown in Table 2. On Kaplan–Meier analysis, patients non-adherent to the ABC pathway had a high risk of MACE (log-rank *p*-value = 0.042) (Figure 4). On the multivariable regression analysis, there was no statistically significant independent association with adverse events (see Table 3).

## Discussion

In real-world anticoagulated AF patients from FANTASIIA registry, we observed a lack of adherence to integrated care management of AF following ABC pathway. The integrated ABC pathway adherent care is associated with a significant reduction in major cardiovascular events during long follow-up, although notable baseline differences between patients with and without ABC-adherent care are present. Control of symptomatology of AF, as assessed by EHRA functional class (B criterion), was associated with the most significant adverse event reductions.

Only 20.2% of the cohort were adherent to the ABC pathway, highlighting the low adherence to integrated care management. These data were consistent with observations from a recent systematic review by Romiti et al. (9) and the study by Pastori et al. (22) in the ATHERO-AF study where only 22.4% of patients were well managed according to the ABC pathway.

According to the “avoid stroke” (i.e., “A” criterion) with adequate oral anticoagulation, only 47% of patients were adherent. To reduce stroke, mortality, and adverse events in AF patients, good anticoagulation with high TTR with VKA or correct dose with DOAC is essential (23, 24). The VKAs have been shown to be highly effective for stroke prevention in AF, reducing the risk of stroke by 64% and all-cause death by 26% compared with control/placebo (25), but the effectiveness of VKA is dependent on the quality of anticoagulation control. Good quality anticoagulation (defined as TTR > 70%) is related with better outcomes during follow-up (26–28). In case of DOACs, those drugs have demonstrated similar or higher efficacy in thromboembolic prevention in AF patients with fixed dose and in the absence of drug monitoring (29–32). However, nearly one-third of patients may receive an inappropriate dose of DOAC in daily clinical practice, according to EHRA recommendations (33), increasing both thromboembolic and bleeding risks (34). Hence, management tools (classical education, smart technology, pill counts…) to improve oral anticoagulation adherence in AF patients involve patients, relatives, and health professionals (physicians, nursery, pharmacist…) (35). Education and counseling to explain the risks of non-adherence are needed to ensure optimal uptake in AF patients with both therapies, improving patient adherence and persistence with treatment (13, 36, 37).

In daily clinical practice, physicians often focus on oral anticoagulation or adverse events or observable risk factors but tend to forget the patient’s perspective, quality of life, and symptomatology related with AF (38). Different studies have analyzed the influence of AF symptoms on quality of life, with assessment tools to quantify the burden of this disease (39). However, the relationship between symptoms and adverse events is not widely analyzed in real-world patients.

In our study, we observed good control of AF symptoms with almost 90% of the sample in EHRA I-II status, and we showed the novel finding that the lack of control of symptoms (B criterion) was the strongest component of the ABC pathway related with adverse events. Such good control of AF symptoms was independently related with a significant reduction in stroke, all-cause mortality, cardiovascular mortality, and MACE. These results were slightly different from the results of ORBIT-AF registry (40) where there was lower quality of life and higher rates of hospitalization in symptomatic patients (EHRA > 2) but with no difference in mortality rates.

We also analyzed the influence of risk factors and comorbidities in AF patients according to an integrated care approach. Risk factor modification, healthy lifestyle, and interventions targeting underlying conditions have shown a reduction in AF burden and recurrence but with a multifaceted approach (12). This “C” criterion definition differs in previous studies, but in the FANTASIIA population, we have selected strict criteria as target control of comorbidities and risk factors according to the current clinical guidelines (Supplementary Figure 1). Hence, only 34.8% of AF patients had risk factors and comorbidities under control. Compared with ESC-EHRA EORP Atrial Fibrillation General Long-Term (AFGen LT) Registry population non-ABC adherent subgroup (41), our patients from FANTASIIA registry were more elderly, with a higher prevalence of cardiovascular or other concomitant diseases, highlighting the high-risk profile of our real-world AF patients. Non-adherence to C criterion had a higher risk, especially for cardiovascular outcomes (MACE, cardiovascular events, and acute myocardial infarction), compared with adherent patients. LaMori et al. (42) analyzed the comorbidity burden of AF patients and observed that almost all (98%) of the population had at least one additional comorbidity, 90% had cardiovascular comorbidities, and ≥ 63% of AF patients had four or more comorbidities in addition to AF, so the high multimorbidity disease burden carried beyond AF status. Indeed, Fauchier et al. (2) analyzed the causes of death in AF patients and only 7% were related with stroke accounting for the majority of deaths related with a cardiovascular cause(s).

In the FANTASIIA registry, only 20% of the AF population were ABC pathway adherent, highlighting the lack of adherence to holistic management of AF patients, despite the potential benefits from adherence to the ABC pathway (9). Stevens et al. (43) recently reviewed 12 clinical studies that focused on the ABC pathway and observed a wide range in the proportion of participants assessed as ABC pathway adherent in the included studies (7.0–43.8%) and near 37% in ATHERO-AF registry (18). In FANTASIIA registry, there was a reduction in MACE/composite outcome in those ABC adherent patients (by 36%) with similar results compared with ATHERO-AF cohort (18) or *post-hoc* AFFIRM trial analysis (44). The recent ESC-EHRA EORP AFGen Long Term registry (41) observed that ABC pathway adherence was associated with a significant lower risk for cardiovascular events, CV death, and all-cause death. Also, in our registry, we observed that the ABC pathway adherent patients had less cardiovascular events, MACE, and clinically significant events (that includes mortality) but no difference in all-cause mortality rates probably related to the small sample sizes of the subgroups.

Compliance with AF management plans, including advice on risk factors and lifestyle changes, simplifying assistance process and prescriptions, better patient education, nurse-led interventions, and close health support, may have a major impact on the burden of AF, improving decision-making, quality of life, and outcomes in AF patients (11, 45). Our study is one of the few based on a prospective cohort and provides more evidence for the use of ABC pathway as the integrated management of AF patients (both under VKA and DOAC), also highlighting the importance of the symptom management. Involving patients, relatives, and multidisciplinary health professionals in the decision-making process is crucial to facilitate truly integrated or holistic management of AF.

### Strengths and Limitations

The limitations of the study are derived from its observational nature, as inclusion bias could not be completely eliminated, although it was minimized by the criterion of consecutive enrollment. However, differences in baseline risk factors characteristics were observed. Unfortunately, we cannot perform a direct comparison using propensity score matching to homogenize the baseline characteristics due to the sample size, so this should be considered a limitation of our study.

Other limitation was related to our use of the robust definition for ABC adherence leading to modest sample sizes of each subgroup with low adverse events and lack of statistical significance for comparison of outcomes, hence limiting the generalizability of the results. Although we have a large follow-up of AF patients, the ABC pathway compliance was calculated at baseline, and the variation of different components of the ABC pathway during the follow-up and the influence on outcomes are uncertain. Moreover, related with the predefined ratio in the protocol, 1:4 for DOACs and VKAs, we could not perform individual analysis to validate ABC pathway for DOAC vs. VKA or for each DOAC due to the small sample size, which is a limitation in the generalizability of the results in the DOAC group.

Regarding the difference between adherence to “A criterion” according to VKA or DOAC therapy, for VKA therapy the definition of good adherence according to TTR 70% is fully accepted in the original definition. However, regarding DOAC definition, there was no uniform approach in previous manuscripts. The most correct definition perhaps will be the assessment of taking DOAC therapy with pharmacy dispensing data from electronic health records, pill count, or laboratory control of DOACs. No studies have previously validated ABC pathway in this way in the DOAC group. However, FANTASIIA registry involves 50 centers in Spain, with different electronic and non-electronic pharmacy dispensing data, and it is not indicated as the laboratory control of DOAC to assess compliance. For that reason, we considered the correct dose of DOAC as an indirect indicator of correct adherence to A criteria, which is a small limitation in the DOAC group of ABC- pathway.

Otherwise, there are several strengths in the FANTASIIA registry, which is a cohort study with prospective long follow-up including a large number of patients with few losses to follow-up, and ≥90% of patients had follow-up data.

## Conclusion

In real-world anticoagulated AF patients from FANTASIIA registry, we observed a lack of adherence to integrated care management of AF following the ABC pathway. AF managed according to the ABC pathway was associated with a significant reduction in adverse outcomes during long follow-up, suggesting the benefit of a holistic and integrated approach to AF management.

## Data Availability Statement

The raw data supporting the conclusions of this article will be made available by the authors, without undue reservation.

## Ethics Statement

The studies involving human participants were reviewed and approved by the Clinical Research Ethics Committee at Hospital Universitario de San Juan (Spain) with the approval number 12/220 and by the Spanish Agency of Medicine and Health Products as a prospective follow-up post-authorization study with the approval number SEC-ACO-2012-01. The patients/participants provided their written informed consent to participate in this study.

## Author Contributions

ME-P and FM drafted the manuscript and writing–review and editing-lead. JM and DO performed the statistical analysis. MA, VB-M, and FM performed as conceptualization-lead, methodology-lead, and writing-review and editing-lead. GL performed the writing–review and editing-supporting. All authors made substantial contributions to the concept and design of the work, participated in the acquisition and interpretation of data, revised the manuscript for important intellectual content, approved the final version of the manuscript, and are able to take public responsibility for the full text of the article.

## Conflict of Interest

DO is employed by ODDS, SL. The remaining authors declare that the research was conducted in the absence of any commercial or financial relationships that could be construed as a potential conflict of interest.

## Publisher’s Note

All claims expressed in this article are solely those of the authors and do not necessarily represent those of their affiliated organizations, or those of the publisher, the editors and the reviewers. Any product that may be evaluated in this article, or claim that may be made by its manufacturer, is not guaranteed or endorsed by the publisher.
