# Supplementary material for: Impact of Integrated Care Management on Clinical Outcomes in Atrial Fibrillation Patients: A Report From the FANTASIIA Registry
Source: Front Cardiovasc Med. 2022 May 2;9:856222. doi: 10.3389/fcvm.2022.856222 (PMC9108173; doi:10.3389/fcvm.2022.856222)
Supplement: Supplementary file 1 [file Data_Sheet_1.docx]

**SUPPLEMENTARY FIGURES**

**Supplementary Figure 1: Definition of each component of ABC Pathway.**

**
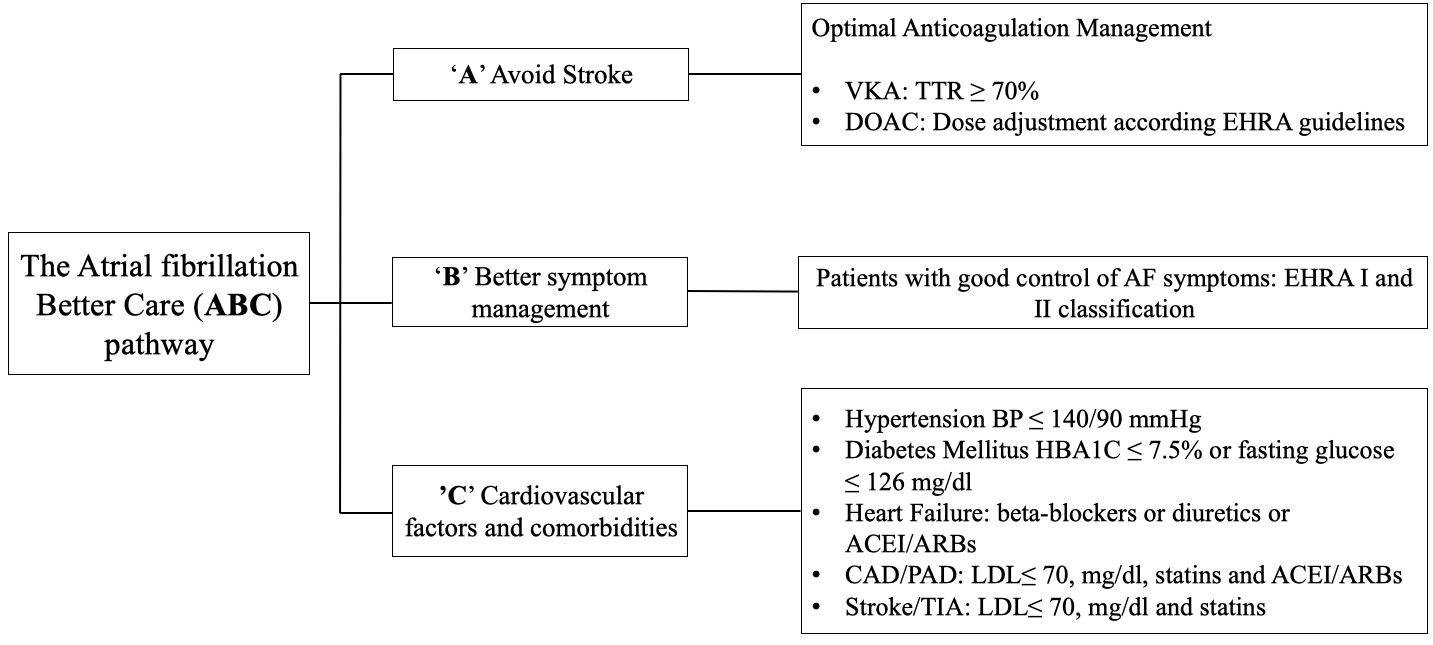
**

**Legend:** VKA: Vitamin K Antagonists. DOAC. Direct Oral Anticoagulants. EHRA: European Heart Rhythm Association. AF: Atrial Fibrillation. BP. Blood pressure. ACEI/ARBs: angiotensin-converting enzyme inhibitors/angiotensin receptor blockers. CAD: coronary artery disease. PAD: peripheral artery disease. TIA: Transitory ischaemic attack. LDL. Low density lipoprotein.

**SUPPLEMENTARY TABLES**

**Supplementary table 1:** Clinical factors related with Cardiovascular Mortality by Univariate and multivariate Cox regression analysis for A component

|  | **Univariate analysis**  **HR (95% CI); p** | **Multivariate analysis**  **HR (95% CI); p** |
| --- | --- | --- |
| Age | 1.09 (1.06-1.12); p<0.001 | 1.08 (1.05-1.11); p<0.001 |
| Male sex | 1.14 (0.78-1.66); p=0.513 | - |
| Diabetes Mellitus | 2.31 (1.58-3.37); p<0.001 | 1.51 (0.96-2.36); p=0.072 |
| Hypertension | 1.43 (0.84-2.43); p=0.188 | - |
| Dyslipidemia | 1.44 (0.97-2.12); p=0.069 | 1.12 (0.74-1.67); p=0.598 |
| ‘A’ criterion fulfilled | 0.59 (0.40-0.88); p=0.009 | **0.67 (0.45-0.99); p=0.048** |
| Coronary artery disease | 2.37 (1.58-3.54); p<0.001 | 1.44 (0.93-2.22); p=0.103 |
| Heart Failure | 3.54 (2.42-5.20); p<0.001 | 2.06 (1.30-3.28); p=0.002 |
| Peripheral Artery Disease | 1.91 (1.04-3.48); p=0.036 | 1.15 (0.59-2.24); p=0.671 |
| Previous stroke/TIA | 1.18 (0.73-1.92); p=0.499 | - |
| Chronic Kidney Disease | 3.53 (2.34-5.32); p<0.001 | 1.70 (1.08-2.66); p=0.021 |
| Charlson Index | 1.61 (1.41-1.83); p<0.001 | 1.28 (1.04-1.57); p=0.019 |

*TIA Transient Ischaemic Attack. HR: Hazard ratio. CI: Confidence interval.*

**Supplementary table 2:** Clinical factors related with All -Cause Mortality by Univariate and multivariate Cox regression analysis for B component

|  | **Univariate analysis**  **HR (95% CI); p** | **Multivariate analysis**  **HR (95% CI); p** |
| --- | --- | --- |
| Age | 1.08 (1.06-1.10); p<0.001 | 1.09 (1.06-1.11); p<0.001 |
| Male sex | 0.70 (0.54-0.90); p=0.006 | 0.72 (0.53-0.99); p=0.044 |
| Diabetes Mellitus | 1.46 (1.13-1.88); p=0.004 | 1.15 (0.84-1.58); p=0.389 |
| Hypertension | 1.66 (1.15-2.39); p=0.007 | 1.48 (0.98-2.24); p=0.061 |
| Dyslipidemia | 1.04 (0.82-1.34); p=0.731 | - |
| ‘B’ criterion fulfilled | 0.31 (0.23-0.42); p<0.001 | **0.49 (0.35-0.69); p<0.001** |
| Coronary artery disease | 1.69 (1.28-2.23); p<0.001 | 1.18 (0.86-1.62); p=0.311 |
| Heart Failure | 2.03 (1.58-2.62); p<0.001 | 1.59 (1.16-2.17); p=0.004 |
| Peripheral Artery Disease | 1.36 (0.88-2.11); p=0.172 | - |
| Previous stroke/TIA | 1.22 (0.90-1.66); p=0.198 | - |
| Chronic Kidney Disease | 2.57 (1.99-3.32); p<0.001 | 1.31 (0.97-1.76); p=0.077 |
| Charlson Index | 1.43 (1.30-1.56); p<0.001 | 1.28 (1.04-1.57); p=0.019 |

*TIA Transient Ischaemic Attack. HR: Hazard ratio. CI: Confidence interval.*

**Supplementary table 3:** Clinical factors related with Cardiovascular Mortality by Univariate and multivariate Cox regression analysis for B component

|  | **Univariate analysis**  **HR (95% CI); p** | **Multivariate analysis**  **HR (95% CI); p** |
| --- | --- | --- |
| Age | 1.08 (1.05-1.11); p<0.001 | 1.08 (1.05-1.12); p<0.001 |
| Male sex | 0.96 (0.65-1.41); p=0.830 | - |
| Diabetes Mellitus | 2.16 (1.48-3.17); p<0.001 | 1.66 (1.02-2.70); p=0.042 |
| Hypertension | 1.35 (0.80-2.31); p=0.265 | - |
| Dyslipidemia | 1.52 (1.03-2.25); p=0.035 | 1.11 (0.74-1.68); p=0.608 |
| ‘B’ criterion fulfilled | 0.19 (0.13-0.30); p<0.001 | **0.39 (0.25-0.62); p<0.001** |
| Coronary artery disease | 2.18 (1.45-3.26); p<0.001 | 1.42 (0.90-2.25); p=0.131 |
| Heart Failure | 2.97 (2.0-4.41); p<0.001 | 2.10 (1.28-3.43); p=0.003 |
| Peripheral Artery Disease | 1.72 (0.94-3.14); p=0.080 | 0.99 (0.50-1.95); p=0.980 |
| Previous stroke/TIA | 1.11 (0.68-1.80); p=0.686 | - |
| Chronic Kidney Disease | 3.15 (2.08-4.76); p<0.001 | 1.53 (0.96-2.44); p=0.072 |
| Charlson Index | 1.56 (1.37-1.78); p<0.001 | 1.29 (1.03-1.62); p=0.025 |

*TIA Transient Ischaemic Attack. HR: Hazard ratio. CI: Confidence interval.*

**Supplementary table 4:** Clinical factors related with MACE by Univariate and multivariate Cox regression analysis for B component

|  | **Univariate analysis**  **HR (95% CI); p** | **Multivariate analysis**  **HR (95% CI); p** |
| --- | --- | --- |
| Age | 1.05 (1.03-1.07); p<0.001 | 1.05 (1.02-1.09); p=0.001 |
| Male sex | 0.78 (0.57-1.06); p=0.110 | 0.91 (0.56-1.48); p=0.710 |
| Diabetes Mellitus | 1.85 (1.36-2.51); p<0.001 | 1.52 (0.90-2.56); p=0.119 |
| Hypertension | 1.41 (0.92-2.15); p=0.115 | 1.20 (0.66-2.21); p=0.550 |
| Dyslipidemia | 1.56 (1.14-2.13); p=0.006 | 1.18 (0.85-1.63); p=0.331 |
| ‘B’ criterion fulfilled | 0.24 (0.17-0.35); p<0.001 | **0.41 (0.28-0.62); p<0.001** |
| Coronary artery disease | 2.56 (1.86-3.51); p<0.001 | 1.82 (1.19-2.79); p=0.006 |
| Heart Failure | 2.27 (1.66-3.10); p<0.001 | 1.76 (1.05-2.97); p=0.033 |
| Peripheral Artery Disease | 1.92 (1.20-3.07); p=0.007 | 1.20 (0.69-2.10); p=0.522 |
| Previous stroke/TIA | 1.37 (0.95-1.98); p=0.096 | 1.29 (0.57-2.92); p= 0.549 |
| Chronic Kidney Disease | 2.16 (1.58-2.95); p<0.001 | 1.32 (0.92-1.90); p=0.127 |
| Charlson Index | 1.45 (1.29-1.61); p<0.001 | 1.15 (0.95-1.40); p=0.157 |

*TIA Transient Ischaemic Attack. HR: Hazard ratio. CI: Confidence interval.*

**Supplementary table 5:** Clinical factors related with Stroke by Univariate and multivariate Cox regression analysis for B component

|  | **Univariate analysis**  **HR (95% CI); p** | **Multivariate analysis**  **HR (95% CI); p** |
| --- | --- | --- |
| Age | 1.01 (0.98-1.05); p=0.475 | - |
| Male sex | 0.64 (0.35-1.18); p=0.153 | - |
| Diabetes Mellitus | 0.93 (0.48-1.77); p=0.817 | - |
| Hypertension | 1.32 (0.59-2.96); p=0.498 | - |
| Dyslipidemia | 1.26 (0.70-2.28); p=0.444 | - |
| ‘B’ criterion fulfilled | 0.21 (0.11-0.43); p<0.001 | **0.28 (0.14-0.59); p<0.001** |
| Coronary artery disease | 1.78 (0.93-3.41); p=0.081 | 1.44 (0.70-2.96); p=0.317 |
| Heart Failure | 1.78 (0.97-3.28); p=0.064 | 1.74 (0.81-3.74); p=0.153 |
| Peripheral Artery Disease | 0.64 (0.15-2.65); p=0.537 | - |
| Previous stroke/TIA | 2.48 (1.33-4.61); p=0.004 | 2.75 (1.20-6.28); p= 0.016 |
| Chronic Kidney Disease | 1.93 (1.06-3.52); p=0.030 | 1.82 (0.93-3.56); p=0.080 |
| Charlson Index | 1.28 (1.02-1.61); p=0.033 | 0.99 (0.71-1.38); p=0.958 |

*TIA Transient Ischaemic Attack. HR: Hazard ratio. CI: Confidence interval.*

**Supplementary table 6:** Baseline characteristic according to B criterion adherence.

| Variable | B criterion Adherence Group  n = 1791 | B criterion non-adherent group  n= 165 | *P value* |
| --- | --- | --- | --- |
| Age | 74.7 ± 9.3 | 77.1 ± 9.7 | <0.001 |
| Sex (female) | 759 (42.3) | 101 (61.2) | <0.001 |
| Comorbidities |  |  |  |
| Hypertension | 1617 (90.2) | 142 (86.1) | 0.171 |
| Diabetes Mellitus | 503 (28.1) | 71 (43.1) | <0.001 |
| COPD | 288 (16.1) | 55 (33.3) | <0.001 |
| PAD | 99 (5.5) | 19 (11.5) | <0.001 |
| CKD | 326 (18.2) | 51 (30.9) | 0.046 |
| Charlson’s Index | 1.3 ± 1.2 | 1.8 ± 1.3 | <0.001 |
| Previous heart disease | 813 (45.3) | 122 (73.9) | <0.001 |
| LVEF | 57.3 ± 11.6 | 58.1±11.5 | 0.045 |
| Hb (g/dL) | 13.5 ± 1.7 | 12.75 ± 1.69 | 0.325 |
| eGFR | 63.1 ± 22.6 | 59.1 ± 21.4 | <0.001 |
| Previous stroke/TIA | 299 (16.7) | 33 (20.1) | 0.088 |
| Previous major bleeding | 72 (4.1) | 8 (4.8) | 0.426 |
| Atrial Fibrillation |  |  |  |
| Paroxysmal AF | 541 (30.2) | 29 (17.6) | <0.001 |
| Persistent AF | 318 (17.6) | 20 (12.1) |  |
| Permanent AF | 858 (47.9) | 107 (64.8) |  |
| Electrical Cardioversion | 302 (16.9) | 27 (16.3) | 0.193 |
| AF ablation | 75 (4.2) | 7 (4.3) | 0.971 |
| Rhythm control | 696 (38.8) | 45 (27.7) | <0.001 |
| Rate control | 1086 (60.6) | 119 (72.3) |  |
| CHA_2_DS_2_-VASc | 3.9 ± 1.5 | 4.7 ± 1.5 | <0.001 |
| HAS-BLED | 2.1 ± 1.1 | 2.5 ± 1.2 | <0.001 |

COPD: Chronic obstructive pulmonary disease. PAD: Peripheral Artery Disease. CAD: Coronary Artery Disease. CKD: Chronic kidney disease. TIA: Transitory Ischemic Attack. LVEF: Left ventricular ejection fraction. BMI (Kg/m^2^): Body mass index**.** Hb: Haemoglobin. ACE: Angiotensin-Converting-Enzyme. ARB: Angiotensin Receptor Blockers. VKA: Vitamin K antagonists. NOACs: Non-vitamin K oral anticoagulants. CHADS_2_ = Congestive heart failure or left ventricular dysfunction (1); Hypertension (1), Age ≥75 (2), Diabetes mellitus (1), prior Stroke/TIA or systemic embolism (2). CHA_2_DS_2_-VASc = Congestive heart failure or left ventricular dysfunction (1); Hypertension (1), Age ≥75 (2) or 65-74 (1), Diabetes mellitus (1), prior Stroke/TIA or systemic embolism (2), Vascular disease (peripheral artery disease, myocardial infarction, aortic plaque) (1), Sex category (i.e. female sex) (1); HAS-BLED = Hypertension (1), Abnormal renal and/or liver function (1), prior Stroke (1), Bleeding history or predisposition (1), Labile INR (1), Elderly (1), Drugs or excess alcohol
