# Supplementary material for: Impact of Integrated Care Management on Clinical Outcomes in Atrial Fibrillation Patients: A Report From the FANTASIIA Registry
Source: Front Cardiovasc Med. 2022 May 2;9:856222. doi: 10.3389/fcvm.2022.856222 (PMC9108173; doi:10.3389/fcvm.2022.856222)
Supplement: Supplementary file 2 [file Data_Sheet_2.docx]

**Appendix**

All investigators of FANTASIIA Registry.

Carmen Rus Mansila^1^, Juan Luis Bonilla Palomas^1^, Antonio Luis Gámez López^1^, Daniel Salas Bravo^1^, Miriam Martín Toro^1^, Antonio Luis Gámez López^1^, Juan Luis Bonilla Palomas^1^, Manuel Anguita Sánchez^2^, Mónica Delgado Ortega^2^, Juan Carlos Castillo Domínguez^2^**,** Martín Ruiz Ortiz^2^, Elías Romo Peña^2^, Mónica Delgado Ortega^2^, Dr. Fátima Esteban^3^, Dr. Miguel Ángel Casanova^3^, Dr. Francisco Torres Calvo^4^, Dr. Rafael Bravo Marqués^4^, Dr. Pedro Chinchurreta Capote^4^, Dr. Alejandro Pérez Cabeza^4^, Dr. Raúl Quirós López^4^, Dr. Rodrigo Mayo Cabeza^4^, Dr. Juan José Gómez Doblas^5^, Dr. José Carlos Pérez Sánchez^5^, Dr. Manuel Almendro Delia^6^, Dr. Cristina Jiménez Hidalgo^6^, Dr. Gonzalo Barón^7^, Dr. Silvia Gómez Moreno^7^, Dr. Antonio Fernández^7^, Dr. Lorena García Riesco^7^. Dr. María Laura García Pérez^8^, Dr. José Rozado Castaño^8^, Dr. Jorge Álvarez^9^, Dr. Tomas Ripoll Vera^9^, Dr. Joan Torres Marqués^9^, Dr. Juana Núñez Morcillo^9^, Dr. Marcos Rodríguez Esteban^10^, Dr. José Alejandro Medina García^10^, Dr. César Sosa^10^, Dr. Javier Mesa Fumero^10^, Dr. Daniel García Fuertes^11^, Dr. Manuel Crespín Crespín^11^, Dr. Inmaculada Coca Prieto^11^, Dr. José Gonzalo Delgado Díaz-Benito^11^, Dr. Elena Villanueva Fernández^11^, Dr. Teresa Cano Mozo^12^, Dr. José Luis Moriñigo Muñoz^12^, Dr. Ana Martín García^12^, Dr. Jesús Manuel Hernández Hernández^12^, Dr. Imanol Otaegui^13^, Dr. Jordi Pérez Rodón^13^, Dr. Jaume Francisco Pascual^13^, Dr. Neus Bellera Gotarda^13^, Dr. Eulalia Roig Minguell^14^, Dra. Sonia Mirabet Pérez^14^, Dr. Vicens Brossa^14^, Dr. Laura López^14^, Dr. Nicolás Manito Lorite^15^, Dr. Ignasi Anguera Camos^15^, Dr. David Chivite Guillén^15^, Dr. Jorge Antonio Morales Álvarez^16^, Dr. Andrés, Fernández Gasalia^16^, Dr. Neus Piulats Egea^16^, Dr. Josep Casas Rodríguez^16^, Dr. Óscar Alcaide^17^, Dr. Roger Villuendas^17^, Dr. Áxel Sarrias^17^, Dr. Mar Domingo^17^, Dr. Ramón de Castro^18^, Dr. Nuria Farré^18^, Dr. Isabel Serrano Rodríguez^18^, Dr. Ingrid Colomer^18^, Dr. José Moreno Arribas^19^, Dr. Vicente Bertomeu Gónzalez^19^, Dr. Bertomeu Martínez^19^, Dr. Francisco Ridocci Soriano^20^, Dr. Rafael Payá Serrano^20^, Dr. Jose Antonio Madrigal Vilata^20^, Dr. José Perez Silvestre^20^, Dr. Juan Cosín Sales^21^, Dr. Francisco Buendía^21^, Dr. Esther Esteban^21^, Dr. Antonio Lillo^21^, Dr. Dolores Marco Macian^21^, Dr. Yolanda Porrás Ramos^22^, Dr. Mª Victoria Mongollón Jiménez^22^, Dr. Pilar Marón Ramos^23^, Dr. Alfonso Varela Román^23^, Dr. Antonio Pose Reino^23^, Dr. Manuel Suárez Tembra^23^, Dr. Manuel Lado López^23^, Dr. Carlos González Juanatey^24^, Dr. Inmaculada Roldán^25^, Dr. Carlos Escobar Cervantes^25^, Dr. Marta Mateos García^25^, Dr. Mª Angustias Quesada Simón^25^, Dr. José Camacho Siles^25^, Dr. Paloma Eviro García^25^, Dr. Manuel Martínez Sellés^26^, Dr. Tomás Datino^26^, Dr. Mª Teresa Vidán^26^, Dr. Luis Cornide^26^, Dr. Pablo García Pavía^27^, Dr. Marta Mª Cobo Marcos^27^, Dr. David Vivas^28^, Dr. Javier Higueres Nafría^28^, Dr. José Manuel Rubio Campal^29^, Dr. Pepa Sánchez Borque^29^, Dr. Francisco Marín Ortuño^30^, Dr. Miguel García Navarro^30^, Dr. Raquel Pérez Luján^30^, Dr. Mariano Leal Hernández^30^, Dr. Sergio Manzano Fernández^30^, Dr. Diego Giménez Cervantes^31^, Dr. Francisco José García Amargo^31^, Dr. Manuel Gonzálvez Ortega^31^, Dr. Milagros Gil Ortega^31^. Dr. Nekane Murga Eizagaechevarría^32^, Dr. Esther Recalde^32^.

**Centers**: ^1^Ciudad de Jaén Hospital, Jaén, Spain. ^2^Reina Sofía Hospital, Córdoba, Spain. ^3^Infanta Margarita Hospital, Cabra, Córdoba, Spain.^4^Costa del Sol Hospital, Marbella, Spain.^5^Virgen de la Victoria Hospital, Málaga, Spain.^6^Virgen Macarena Hospital, Sevilla, Spain. ^7^Virgen del Rocío Hospital, Seviila, Spain. ^8^Central de Asturias Hospital, Asturias, Spain. ^9^Son Llázer Hospital, Baleares, Spain. ^10^Candelaria Hospital, Canarias, Spain. ^11^Santa Bárbara Hospital, Ciudad Real, Spain. ^12^Clínico de Salamanca Hospital, Salamanca, Spain. ^13^Vall d’Hebrón Hospital, Barcelona, Spain. ^14^Sant Pau Hospital, Barcelona, Spain.^15^Universitari de Bellvitge Hospital, Barcelona, Spain. ^16^Del Mar Hospital, Barcelona, Spain. ^17^Germans Trias, Barcelona, Spain. ^18^Juan XXIII, Tarragona, Spain. ^19^San Juan de Alicante Hospital, San Juan, Spain. ^20^General Hospital, Valencia, Spain. ^21^Arnau de Vilanova Hospital, Valencia, Spain. ^22^ Cáceres Hospital, Cáceres, Spain. ^23^Clínico de Santiago de Compostela, Galicia, Spain. ^24^Universitario Lucus Augusti Hospital, Galicia, Spain. ^25^La Paz Hospital, Madrid, Spain. ^26^Gregorio Marañon Hospital, Madrid, Spain. ^27^Puerta de Hierro Hospital, Madrid, Spain.^28^Clínico San Carlos, Madrid, Spain. ^29^Fundación Jiménez Díaz Hospital, Madrid, Spain. ^30^Virgen de la Arrixaca Hospital, Murcia, Spain. ^31^Morales Meseguer Hospital, Murcia, Spain. ^32^Basurto Hospital, Vizcaya, Spain
